# Felt Versus Pericardium for the Sandwich Technique in Type A Aortic Dissection: A Human Cadaver Study

**DOI:** 10.3390/jcm14217736

**Published:** 2025-10-31

**Authors:** Jasmine El-Nashar, Thomas Poschner, Mohamed El Din, Paata Pruidze, Giorgi Didava, Amila Kahrovic, Wolfgang J. Weninger, Daniel Zimpfer, Marek P. Ehrlich, Emilio Osorio-Jaramillo

**Affiliations:** 1 Center for Anatomy and Cell Biology, Division of Anatomy, Medical University of Vienna, 1090 Vienna, Austria; 2 Department of Cardiac and Thoracic Aortic Surgery, Medical University of Vienna, 1090 Vienna, Austria

**Keywords:** aortic dissection, aortic repair, DANE, cardiac surgery

## Abstract

**Background:** The *Sandwich* technique is a commonly adopted method for reinforcing the dissected aortic wall during acute Type A aortic dissection (ATAAD) repair, using either felt or bovine pericardial strips. However, complications such as anastomotic bleeding, distal anastomotic new entry (DANE) and persistent false lumen (PFL) remain major challenges. This study evaluated and compared the sealing efficacy of felt versus pericardium in a human cadaver model. **Methods:** ATAAD was simulated in 20 fresh human cadavers. Repairs were performed using the *sandwich* technique with either felt (n = 10) or pericardium (n = 10), followed by end-to-end prosthetic graft anastomosis. Procedure time was recorded. Following the repair, the aortas were perfused at 160/90 mmHg using a glycerol-water solution to assess fluid leakage (mL), DANE and PFL. **Results:** Median leakage was significantly lower in the pericardium group (67.5 mL [IQR 40–198.8]) compared to the felt group (315 mL [IQR 285–445], *p* = 0.002). Procedure times were comparable between groups. DANE occurred in 20% (pericardium) and 30% (felt) of cases, while PFL was observed in 30% of cases in both groups; differences were not statistically significant. **Conclusions:** The superior sealing properties of pericardium in this study suggest a promising approach for reducing leakage in ATAAD repair. While rates of DANE and PFL were comparable, the advantage of pericardium was confined to leakage reduction. These findings highlight the need for further research to determine whether this experimental benefit translates into improved clinical outcomes.

## 1. Introduction

Acute Type A aortic dissection (ATAAD) is a life-threatening emergency that requires immediate surgical intervention [1]. Surgical replacement of the ascending aorta with reinforcement of the distal anastomosis remains the standard treatment. However, achieving a secure and hemostatic distal anastomosis is technically challenging. Bleeding from needle holes or anastomotic mismatch can lead to serious perioperative and long-term complications, contributing to significant morbidity and mortality even years after the repair [2,3,4,5].

A major concern in ATAAD repair is the persistence of the false lumen (PFL), which may result from inadequate reapproximation of the aortic wall layers, incomplete suture apposition, or the development of a new distal entry tear—referred to as distal anastomotic new entry (DANE) [2,6]. Both DANE and PFL increase the risk of ongoing false lumen pressurization, aortic expansion, reintervention, and reduced long-term survival [6].

Various reinforcement strategies have been proposed, including the use of Teflon felt or pericardial strips [7,8], BioGlue fixation [9], adventitial inversion [10], stent graft approaches [11], and various modifications thereof. Among these, the double patch “*sandwich* technique” is one of the most widely used. This method involves placing two strips of reinforcement material—typically Teflon felt or pericardium—on both the outer and inner surfaces of the aortic wall to minimize bleeding and reduce the risk of new entry tears during suturing, followed by implantation of a vascular prosthesis [12]. However, more recent evidence suggests that a surgeon’s expertise and meticulous surgical technique may be the most critical determinants influencing rates of DANE and PFL, rather than the reinforcement material itself. Notably, the use of Teflon felt or biological glue—compared with no reinforcement material—has not consistently demonstrated superiority in preventing these complications [13].

Despite this, the optimal choice of reinforcement material remains a subject of debate due to differences in mechanical properties, tissue integration, and handling characteristics. To address this gap, our study compares these two materials in a standardized human cadaver model of ATAAD repair, aiming to provide experimental evidence that may inform surgical decision-making and improve clinical outcomes.

## 2. Materials and Methods

**Figure 1:**
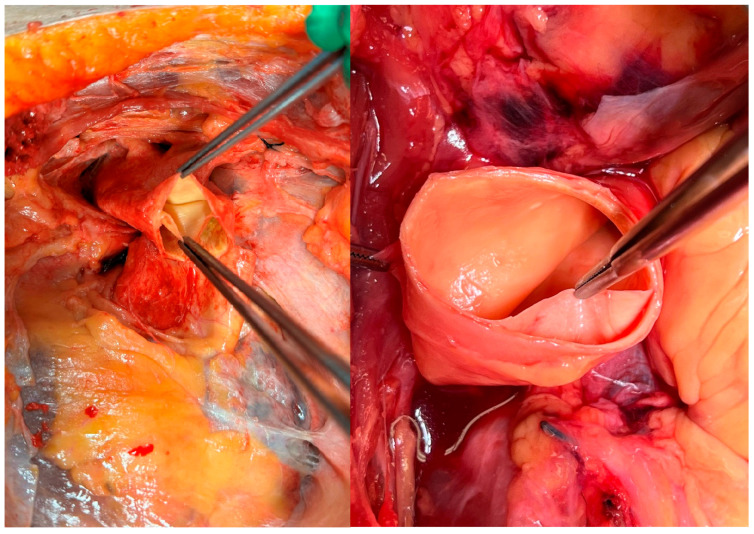
Artificially created aortic dissection.

Twenty fresh human cadavers with artificially created type A aortic dissections (Figure 1) underwent surgical repair using the *sandwich* technique with either felt or pericardial strips. Only cadavers with a post-mortem interval of less than 72 h and no prior history of thoracic aortic surgery were included. The study was approved by the Ethics Committee of the Medical University of Vienna (No. 1997/2024). All donors had provided informed consent during their lifetime for body donation for physician education, continuing medical training, and medical research. This consent was verified by an additional confirmation letter from the institutional body donation program, which was submitted to the journal.

Ten procedures used felt strips for the *sandwich* technique, while the remaining ten utilized pericardial strips. The time required to complete the *sandwich* technique and prosthetic graft anastomosis was recorded in minutes (min) and seconds (s). Leakage volume, simulating blood loss, was measured in milliliters (mL). The integrity of the *sandwich* technique and anastomosis was assessed by identifying complications such as persistent false lumen (PFL) and distal anastomotic new entry (DANE). Perfusion was maintained at a standardized pressure of approximately 160/90 mmHg. To evaluate potential factors influencing the outcomes of the simulated surgical procedure, demographic information and medical histories of the cadavers were recorded.

### 2.1. Cadaver Model Preparation and Setup

Surgical access was obtained through a median sternotomy. The pericardium was opened, and the ascending aorta along with its branch vessels, was carefully exposed and mobilized. A transverse aortotomy was performed approximately 2 cm proximal to the brachiocephalic trunk. An artificial dissection was created by separating the intimal layer from the medial layer of the aortic wall with an 11-blade scalpel and a dissector. The dissection was subsequently extended approximately 1.5 cm distally toward the aortic arch and widened in a semicircular fashion using a dissector.

### 2.2. Surgical Repair and Model Perfusion

**Figure 2:**
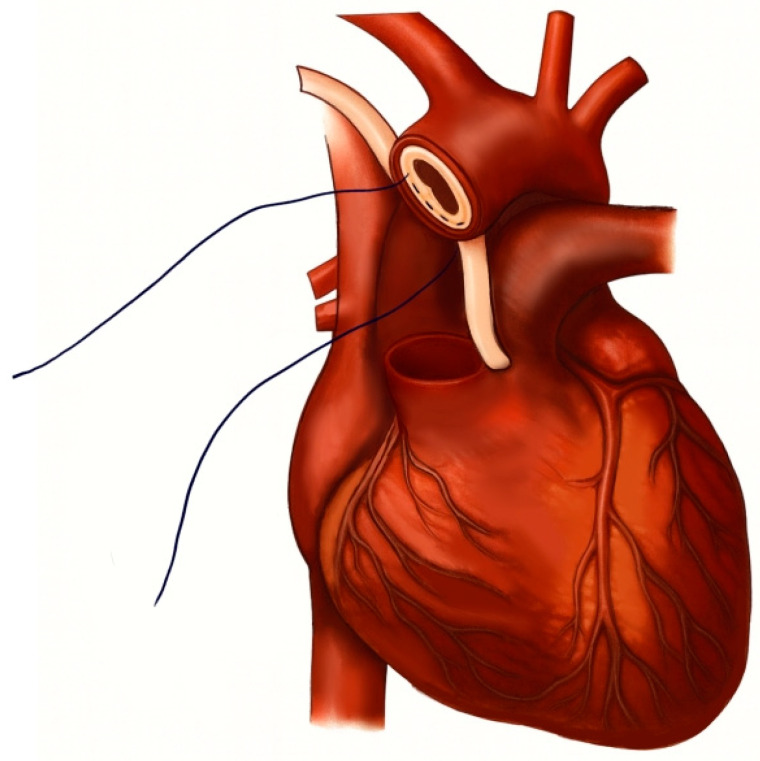
Schematic illustration of the *sandwich* technique.

**Figure 3:**
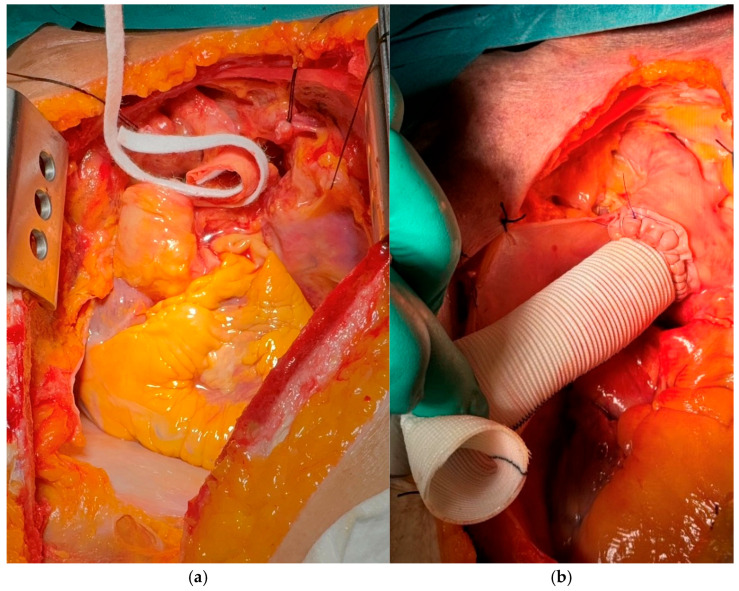
Intraoperative view of the *sandwich* technique for aortic repair. (**a**) Reinforcement of the dissected aortic wall using dual strips of pericardium or synthetic felt. (**b**) Completed anastomosis of the aortic prosthesis to the reinforced aortic stump.

Aortic repair was performed by reapproximating the dissected aortic wall layers using the *sandwich* technique, followed by implantation of a vascular graft. For the *sandwich* technique, two strips—either felt or pericardium—approximately 7 mm in width were prepared, with their lengths adjusted to match the specific diameter of the aorta (see schematic representation in Figure 2 and Figure 3a,b). The structural and mechanical characteristics of the two reinforcement materials are summarized in Table 1. The primary strip was positioned externally to encircle the aortic wall, while the secondary strip was placed on the inner/luminal surface. The dissected aortic wall layers were then secured between the two strips using a horizontal mattress suture with 4-0 polypropylene, ensuring proper reapproximation of the tissue layers. Subsequently, an aortic replacement graft was anastomosed to the reinforced aortic stump in an end-to-end fashion with a continuous running suture (Figure 3b). Finally, the aortic arch was resected just proximal to the origins of the left common carotid and subclavian arteries, corresponding to zone 1 according to the Ishimaru classification [14].

Following surgical repair, each specimen underwent both static and dynamic perfusion using a mechanical pump to evaluate the integrity of the aortic repair. The pump was programmed to deliver a pulsatile flow pattern consisting of a 2 s active flow phase followed by a 5 s pause. During the active phase, the flow rate was maintained at 100 mL/s, corresponding to an average overall flow rate of 1 L/min. Each perfusion cycle was conducted for a total duration of 20 min per simulation. A fluid column generating a diastolic pressure of 90 mmHg was used to replicate physiological condition, while systolic pressure was regulated and maintained at 160 mmHg using an external pressure monitoring system.

**Figure 4:**
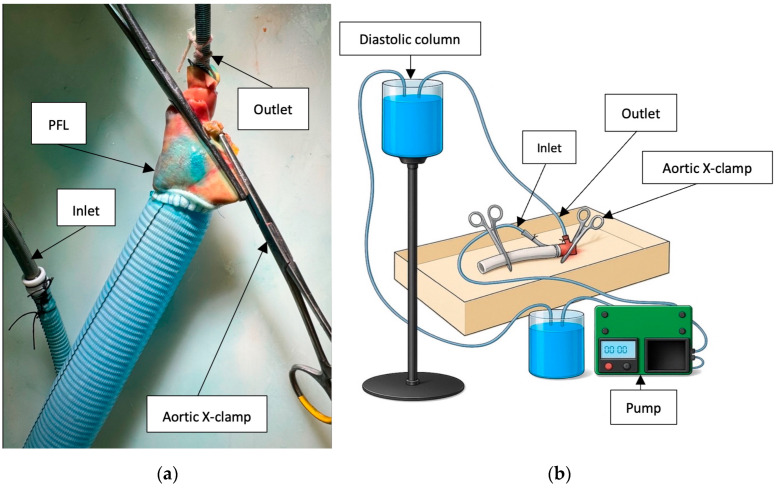
Perfusion assessment of aortic repair in the artificial dissection model, performed according to the experimental setup described in reference [15]. (**a**) Visualization of PFL, as indicated by blue dye staining within the false lumen. (**b**) Experimental setup used to simulate ATAAD repair.

The inlet ECMO cannula was inserted into the side branch of the aortic prosthesis, and the outlet ECMO cannula was positioned within the brachiocephalic trunk. Both cannulas were securely ligated to maintain a closed circulatory circuit. Large vascular cross-clamps were applied to the distal end of the prosthesis and the native aortic arch (Figure 4a,b).

**Figure 5:**
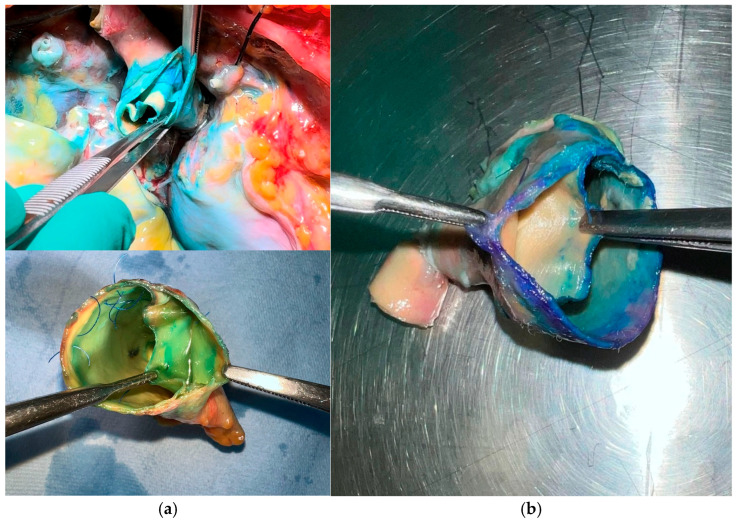
Post-perfusion assessment of aortic repair in an artificial dissection model. (**a**) PFL, indicted by blue dye staining within the false lumen, reflecting incomplete sealing. (**b**) Successful sealing with no PFL.

To simulate blood viscosity, a fluid mixture consisting of 40% glycerol and 60% water was used. The solution was stained with methylene blue at a concentration of 16 mL/L to enhance visualization of fluid leakage, PFL (Figure 5a) and DANE [15].

### 2.3. Evaluation of Integrity of Sandwich Technique and Vascular Graft Anastomosis

The explanted aorta was placed in an appropriate container to enable collection and quantification of fluid loss. Prior to the 20 min perfusion phase, leakage sites were identified, and additional sutures were applied as required. To assess iatrogenic dissection, all sutures were carefully removed following perfusion. The presence of new intimal tears and persistent false lumen was evaluated by detecting blue staining between the dissected layers (Figure 5a).

### 2.4. Statistics

Continuous variables were expressed as medians with interquartile ranges (IQRs) and categorical variables as frequencies and percentages. Group comparisons for continuous variables were performed using the Mann–Whitney U test, and Fisher’s exact test was applied for categorical variables. The composite endpoint was defined as the occurrence of either distal anastomotic new entry (DANE) or persistent false lumen (PFL). Statistical significance was assessed at a two-sided α level of 0.05. All statistical analyses were performed using SPSS version 29 (IBM Corp., Armonk, NY, USA).

## 3. Results

### 3.1. Demographic Characteristics and Anatomical Findings of Study Specimens

The study included 20 body donors with a median age of 82 years (IQR 72–86). The overall proportion of female donors was 50%, with 70% in the pericardium group and 30% in the felt group, without statistical significance (*p* = 0.179). Aortic calcification at the suture site was observed in 50% of cases overall, equally distributed between the pericardium and felt group, with 5 cases each (*p* = 1.000). Calcification was categorized as follows: absent in 10 (50%) cases (pericardium: 5 [50%]; felt: 5 [50%]), mild/moderate in 6 (30%) cases (pericardium: 3 [30%]; felt: 3 [30%]), and severe in 4 (20%) cases (pericardium: 2 [20%]; felt: 2 [20%]; *p* = 1.000). The median aortic diameter was 29 mm (27.3–31.5) overall, 28.5 mm (26.8–32.5) in the pericardium group, and 29.5 mm (27.5–30.5) in the felt group (*p* = 0.912). The median aortic thickness was overall 1.3 mm (1.2–1.6), in the pericardium group 1.3 mm (1.1–1.5), and 1.3 mm (1.2–1.7) in the felt group (*p* = 0.280) (Table 2).

Appendix A provides details on anatomical findings (Table A1), the donors cause of death and their concomitant diseases (Table A2), the time needed for the *sandwich* procedure and prosthesis implantation (Table A3), the number of stitches for the *sandwich* procedure and prosthesis implantation (Table A4), the cases of distal anastomotic new entry and persistent false lumen (Table A5) and the location of iatrogenic dissection, location of calcification and location of intima tear (Table A6).

### 3.2. Procedural Outcomes

Overall, the median time for the *sandwich* technique was 14:02 min (12:36–15:56) minutes, with 15:22 min (12:36–18:45) in the pericardium group and 13:00 min (11:14–14:47) in the felt group (*p* = 0.089). The median time for prosthesis implantation was 17:12 min (14:12–20:10) overall, 17:35 min (14:42–21:38) in the pericardium group, and 16:31 min (13:37–19:36) in the felt group (*p* = 0.247). The median number of sutures for the *sandwich* technique was 20 (18–22) combined, with 20 (18–22) in the pericardium group and 20 (18–23) in the felt group (*p* = 0.796). For prosthesis anastomosis, the median number of sutures was 26 (24–28) overall, 27 (24–29) in the pericardium group, and 25 (23–30) in the felt group (*p* = 0.529).

The median number of additional U-sutures was 1 (0–2) overall, with 2 (0–2) in the pericardium group and 1 (0–2) in the felt group (*p* = 0.393). The median leakage volume was 240 mL (58.8–327.5 mL) in the total group, 67.5 mL (40–198.8 mL) in the pericardium group, and 315 mL (285–445 mL) in the felt group (*p* = 0.002).

DANE was observed in 5 (25%) cases overall, 2 (20%) cases in the pericardium group, and 3 (30%) cases in the felt group (*p* = 1.000). PFL was noted in 6 (30%) cases overall, 3 (30%) cases in the pericardium group, and 3 (30%) cases in the felt group (*p* = 1.000). In the pericardium group, a DANE was observed in two cases. In one case, the tear was located at the suture site within an area of dissection and calcification. In the second case, the tear occurred in a region of dissection and calcification but was not located at the suture site. The composite endpoint, defined as DANE or PFL, had a median value of 0 (0–2) in the total group, 0 (0–1) in the pericardium group, and 0 (0–2) in the felt group (*p* = 0.912) (Table 3).

## 4. Discussion

This study is, to our knowledge, the first to directly compare felt and pericardium for anastomotic sealing in an experimental model. The use of pericardium as a *sandwich* material resulted in a significantly lower leakage volume compared to synthetic felt. Despite its superior sealing performance, no measurable differences were observed in the occurrence of DANE, PFL, or the composite endpoint.

Optimization of the proximal and distal anastomosis has been a major focus in aortic surgery for several decades. While the *sandwich* technique with Teflon felt remains the standard [5], alternative methods such as the adventitia inversion technique have been described in the surgical literature to further optimize hemostasis [16]. Additionally, techniques using tissue glue have been explored for several decades; although they may improve local hemostasis, they have not consistently reduced reintervention rates [17]. Furthermore, concerns regarding stroke have led some centers to avoid the routine use of these methods [18].

A perfectly sealed distal anastomosis is particularly crucial, as inadequate closure can potentially lead to serious complications. The occurrence of DANE or PFL exposes patients to immediate risks such as malperfusion of vital organs, and long-term consequences including progressive aneurysmal disease, both of which increase the likelihood of reintervention. Another important concern during aortic repair is hemorrhage arising from suture needle tracts or from insufficient sealing at the aortic-prosthetic interface, potentially resulting in considerable intraoperative blood loss. Therefore, even minor improvements in sealing quality may translate into significantly better short- and long-term outcomes.

The significantly reduced leakage volume with pericardium may be explained by the inherent material properties. Reasons may be the lower porosity of pericardium as compared to felt, as well as the superior pliability of the biological material, which may allow for a more uniform distribution of suture tension and better adaptation to the aortic wall, thereby potentially enhancing local hemostasis and seal integrity. While these findings did not correspond to measurable differences in clinically relevant surrogate parameters such as DANE, PFL, or intimal tears, improved sealing could nonetheless reduce intraoperative bleeding and transfusion requirements. This potential clinical advantage, however, remains to be confirmed in further studies.

A possible explanation for the lack of clinical benefit may also lie in the multifactorial nature of anastomosis complications. Anatomical factors such as aortic wall quality or the presence of calcifications, as well as surgical factors including needle blunting after repeated penetration or technical difficulties with the anastomosis itself, could play a crucial role in the occurrence of adverse events, making the choice of buttressing material not the sole determinant [13,19,20]. Additionally, as emphasized by Khonsari, it is equally “important […] to provide appropriate tension on the suture line” to prevent bleeding from loose suture lines or intimal tears from improper suture handling [19].

The use of pericardial strips is likely associated with slightly greater procedural complexity, as the softer and more elastic nature of pericardium, compared with the stiffer felt, makes it more challenging to handle and suture. This is reflected by somewhat longer anastomotic times, although the difference was not statistically significant. Whether this discrepancy has clinical relevance remains unclear. Nevertheless, this finding highlights the importance of simulation and training—such as on cadaver models—in cardiac surgery, especially for low-volume centers [15,21]. For example, in the present study, a significant improvement was observed between the first four and the last four *sandwich* procedures with pericardium (*p* = 0.029).

Several limitations must be acknowledged, which may affect the generalizability of the results. The small sample size (n = 10 per group) limits statistical power, particularly for detecting differences in outcomes such as DANE and PFL. This was mainly due to the restricted availability of body donors. Moreover, the experimental setup cannot fully replicate in vivo conditions, as it lacks coagulation, tissue repair, prolonged vascular remodeling and realistic dissection mechanisms. The glycerol solution only mimics blood viscosity without the cellular and hemostatic components required for physiological healing. Finally, long-term effects such as re-operations or clinical outcomes cannot be assessed with this model.

## 5. Conclusions

This study represents the first comparative investigation of pericardium versus felt in a standardized human cadaver model of Type A aortic dissection repair. Pericardium demonstrated superior immediate sealing properties but no measurable advantage regarding DANE or PFL, limiting its benefit to leakage reduction. While it may enhance local hemostasis, these findings provide a foundation for future research to determine whether this experimental benefit translates into clinical improvement.

## Figures and Tables

**Table 1:** Comparison of pericardium and felt as reinforcement materials in the *sandwich* technique for aortic dissection repair.

Characteristics	Pericardium	Felt
Material type	biological tissue (autologous or bovine pericardium)	synthetic polymer (non-absorbable)
Biocompatibility	integrates with native tissue; minimal inflammatory response	inert and stable; minimal foreign body reaction
Pliability	soft and flexible; conforms well to vessel contours	stiffer; maintains flat shape
Stiffness	compliant and elastic	rigid and supportive
Handling	easy to trim, but may twist or adhere to native tissue	easy to handle and position during suturing
Suture adaptability	smooth needle passage; tension evenly distributed	higher resistance to needle passage; higher suture-line tension
Hemostatic effect	seals suture holes naturally; biologically promotes coagulation	provides strong mechanical sealing
Seal integrity	pliable surface conforms closely to the aortic wall and prosthesis, minimizing micro-gaps and bleeding sites	stiffer structure may leave small interfacial gaps, particularly on irregular surfaces

**Table 2:** Demographic and morphometric characteristics of study specimen.

		Overall	Pericardium Group	Felt Group	*p*-Value
Age, years, median (IQR)		82 (72–86)	83 (77–89)	77 (68–83)	0.089 *
Gender, n (%)	female	10 (50%)	7 (70%)	3 (30%)	0.179 **
	male	10 (50%)	3 (30%)	7 (70%)	
Aortic calcification at suture site, n (%)	none	10 (50%)	5 (50%)	5 (50%)	
	mild/moderate	6 (30%)	3 (30%)	3 (30%)	
	severe	4 (20%)	2 (20%)	2 (20%)	1.000 ***
Aortic calcification at suture site, n (%)	Yes	10 (50%)	5 (50%)	5 (50%)	1.000 **
	No	10 (50%)	5 (50%)	5 (50%)	
Aortic diameter (mm), median (IQR)		29 (27.3–31.5)	28.5 (26.8–32.5)	29.5 (27.5–30.5)	0.912 *
Aortic thickness (mm), median (IQR)		1.3 (1.2–1.6)	1.3 (1.1–1.5)	1.3 (1.2–1.7)	0.280 *
Aortic thickness (mm) at 12 o’clock, median (IQR)		1.2 (1–1.5)	1.2 (1–1.3)	1.4 (1.2–1.7)	0.123 *
Aortic thickness (mm) at 12–3 o’clock clockwise, median (IQR)		1.3 (1–1.6)	1.3 (0.8–1.5)	1.4 (1.1–1.7)	0.315 *
Aortic thickness (mm) at 3 o’clock, median (IQR)		1.3 (1.1–1.5)	1.2 (1–1.5)	1.4 (1.2–1.5)	0.165 *
Aortic thickness (mm) at 3–6 o’clock clockwise, median (IQR)		1.4 (1.2–1.7)	1.3 (1–1.8)	1.5 (1.2–1.7)	0.481 *
Aortic thickness (mm) at 6 o’clock, median (IQR)		1.5 (1.2–1.8)	1.4 (1.1–1.6)	1.6 (1.4–1.8)	0.315 *
Aortic thickness (mm) at 6–9 o’clock clockwise °, median (IQR)		1.3 (1.1–1.6)	1.2 (1.1–1.5)	1.5 (1.1–1.8)	0.393 *
Aortic thickness (mm) at 9 o’clock, median (IQR)		1 (1–1.4)	1.05 (0.9–1.5)	1 (1–1.5)	0.796 *
Aortic thickness (mm) at 9–12 o’clock clockwise, median (IQR)		1.25 (1–1.5)	1.2 (1–1.4)	1.4 (1–1.5)	0.280 *

Values are n (%). * = Mann–Whitney U test, ** = Fisher’s exact test, *** = Pearson’s chi-squared test. IQR, interquartile range.

**Table 3:** Surgical and perfusion test outcomes.

		Overall	Pericardium Group	Felt Group	*p*-Value
Time *sandwich* minutes, median (IQR)		14:02 (12:36–15:56)	15:22 (12:36–18:45)	13:00 (11:14–14:47)	0.089 *
Time prosthesis minutes, median (IQR)		17:12 (14:12–20:10)	17:35 (14:42–21:38)	16:31 (13:37–19:36)	0.247 *
Sutures *sandwich*, median (IQR)		20 (18–22)	20 (18–22)	20 (18–23)	0.796 *
Sutures prosthesis, median (IQR)		26 (24–28)	27 (24–29)	25 (23–30)	0.529 *
Add on U-sutures, median (IQR)		1 (0–2)	2 (0–2)	1 (0–2)	0.393 *
Add on Z-sutures, median (IQR)		0 (0–0)	0 (0–0)	0 (0–0)	0.481 *
Leakage (mL), median (IQR)		240 (58.8–327.5)	67.5 (40–198.8)	315 (285–445)	0.002 *
DANE, n (%)	Yes	5 (25%)	2 (20%)	3 (30%)	1.000 **
	No	15 (75%)	8 (80%)	7 (70%)	
Intima tear size (mm), median (IQR)		0 (0–0.8)	0 (0–0.3)	0 (0–1.3)	0.684 *
PFL, n (%)	Yes	6 (30%)	3 (30%)	3 (30%)	1.000 **
	No	14 (70%)	7 (70%)	7 (70%)	
Composite endpoints, median (IQR)		0 (0–2)	0 (0–1)	0 (0–2)	0.912 *

Values are n (%). * = Mann–Whitney U test, ** = Fisher’s exact test; DANE, distal anastomotic new entry; PFL, persistent false lumen; IQR, interquartile range.

## Data Availability

The data presented in this study are available on request from the corresponding author.

## Abbreviations

The following abbreviations are used in this manuscript:

ATAAD	Acute Type A aortic dissection
CAD	Coronary artery disease
COPD	Chronic obstructive pulmonary disease
CPR	Cardiopulmonary resuscitation
DANE	Distal anastomotic new entry
HTN	Arterial hypertension
IQR	Interquartile range
min	Minutes
mL	Milliliters
mm	Millimeters
PFL	Persistent false lumen
RBBB	Right bundle branch block
s	Seconds
T2DM	Type 2 diabetes mellitus

## Appendix A

**Table A1:** Anatomical findings.

Group	Ascending Aortic Calcification	Aortic Calcification at Suture Site *	Location of Aortic Calcification on Suture Site (Clockwise Orientation)
**Pericardium Group**			
Body donor 1	mild/moderate	Yes	10–12 o’clock
Body donor 2	mild/moderate	Yes	2–4 o’clock
Body donor 3	none	No	None
Body donor 4	severe	Yes	3–9 o’clock
Body donor 5	none	No	None
Body donor 6	none	No	None
Body donor 7	mild/moderate	Yes	5–8 o’clock
Body donor 8	severe	Yes	1–4 o’clock 7 o’clock
Body donor 9	none	No	None
Body donor 10	none	No	None
**Felt Group**			
Body donor 1	none	No	None
Body donor 2	mild/moderate	Yes	3–6 o’clock
Body donor 3	none	No	None
Body donor 4	none	No	None
Body donor 5	mild/moderate	Yes	3 o’clock
Body donor 6	none	No	None
Body donor 7	none	No	None
Body donor 8	mild/moderate	Yes	5 o’clock
Body donor 9	severe	Yes	4–5 o’clock
Body donor 10	severe	Yes	12 o’clock

* Aortic calcification at the suture site was systematically mapped in a clockwise orientation, with the 12 o’clock position corresponding to the brachiocephalic trunk.

**Table A2:** Donor Characteristics.

Group	Cause of Death	Concomitant Diseases
**Pericardium Group**		
Body donor 1	cardiac decompensation	none
Body donor 2	mammary carcinoma	none
Body donor 3	terminal disease progression in Roussey-Levy syndrome	decubitus
Body donor 4	cardiac decompensation	HTN
Body donor 5	cardiac decompensation	myelodysplastic syndrome, pancytopenia, myocardial infarction
Body donor 6	multi-organ failure	polytrauma after window fall, small-bowel perforation, splenic rupture
Body donor 7	senile marasmus	cardiac failure, mitral valve insufficiency, aortic valve insufficiency, HTN
Body donor 8	kidney failure	none
Body donor 9	central pulmonary embolism	status post CPR, HTN, status post cerebral hemorrhage
Body donor 10	exacerbated COPD	emphysema, HTN, T2DM, RBBB
**Felt Group**		
Body donor 1	septic multi-organ failure	laryngeal carcinoma, metastases, neutropenia
Body donor 2	respiratory global insufficiency, pneumonia	severe COPD, HTN, T2DM, ischemic cardiomyopathy, hyperlipidemia
Body donor 3	multiorgan failure	sepsis, chronic hip infection
Body donor 4	pneumonia	epilepsy, T2DM
Body donor 5	myocardial infarction	T2DM, HTN, atrial fibrillation
Body donor 6	primary metastatic cholangiocellular adenocarcinoma	cardiac failure, CAD, HTN, aortic valve stenosis
Body donor 7	unknown	unknown
Body donor 8	pulmonary heart disease	COPD
Body donor 9	bronchial carcinoma	central lession of the lung, COPD
Body donor 10	liver failure	cholangiocarcinoma, liver cirrhosis Child-Pugh A

HTN = arterial hypertension, CPR = cardiopulmonary resuscitation, T2DM = type 2 diabetes mellitus, RBBB = right bundle branch block, CAD = coronary artery disease, COPD = chronic obstructive pulmonary disease.

**Table A3:** Time for *sandwich* procedure and prosthesis implantation.

Group	Time *Sandwich* Minutes	Time Prosthesis Minutes
**Pericardium Group**		
Body donor 1	20:26	14:08
Body donor 2	22:17	17:05
Body donor 3	14:20	16:06
Body donor 4	12:36	14:24
Body donor 5	15:07	14:49
Body donor 6	17:03	20:45
Body donor 7	15:38	20:16
Body donor 8	18:12	24:18
Body donor 9	12:36	24:53
Body donor 10	10:53	18:05
**Felt Group**		
Body donor 1	16:03	13:58
Body donor 2	14:46	17:20
Body donor 3	12:44	14:01
Body donor 4	13:44	12:35
Body donor 5	10:56	11:08
Body donor 6	14:52	18:12
Body donor 7	12:36	21:05
Body donor 8	11:21	19:30
Body donor 9	13:17	19:55
Body donor 10	09:10	15:43

**Table A4:** Number of stitches for *sandwich* procedure and prosthesis implantation.

Group	Stitches *Sandwich*	Stitches Prosthesis	Leakage (mL)
**Pericardium Group**			
Body donor 1	20	24	240
Body donor 2	20	30	40
Body donor 3	20	27	40
Body donor 4	22	27	85
Body donor 5	18	23	15
Body donor 6	26	28	660
Body donor 7	23	25	40
Body donor 8	20	31	185
Body donor 9	18	24	90
Body donor 10	14	26	50
**Felt Group**			
Body donor 1	18	25	550
Body donor 2	18	21	200
Body donor 3	20	28	310
Body donor 4	24	24	300
Body donor 5	16	23	330
Body donor 6	27	27	750
Body donor 7	22	34	310
Body donor 8	20	35	410
Body donor 9	20	24	240
Body donor 10	22	22	320

**Table A5:** Cases of distal anastomotic new entry (DANE) and persistent false lumen (PFL).

Group	DANE	Size of Intimal Tear (mm)	PFL
**Pericardium Group**			
Body donor 1	No		No
Body donor 2	Yes	1	Yes
Body donor 3	No		No
Body donor 4	No		No
Body donor 5	No		No
Body donor 6	No		Yes
Body donor 7	Yes	2	Yes
Body donor 8	No		No
Body donor 9	No		No
Body donor 10	No		No
**Felt Group**			
Body donor 1	No		No
Body donor 2	Yes	3	Yes
Body donor 3	No		No
Body donor 4	No		No
Body donor 5	No		No
Body donor 6	No		No
Body donor 7	Yes	2	Yes
Body donor 8	No		No
Body donor 9	No		No
Body donor 10	Yes	2	Yes

**Table A6:** Location of iatrogenic dissection, location of calcification and location of intima tear.

Group	Location of Iatrogenic Dissection	Location of Calcification	Location of New Intima Tear
**Pericardium Group**			
DANE 1	12–6 o’clock	2–4 o’clock	3 o’clock
DANE 2	12–6 o’clock	5–8 o’clock	6 o’clock
**Felt Group**			
DANE 1	6–1 o’clock	3–6 o’clock	5 o’clock
DANE 2	12–6 o’clock		3 o’clock
DANE 3	6–12 o’clock	12 o’clock	6 o’clock

Location of iatrogenic dissection, calcification and intima tear was systematically mapped in a clockwise orientation, with the 12 o’clock position corresponding to the brachiocephalic trunk.
